# Using earth mover’s distance for viral outbreak investigations

**DOI:** 10.1186/s12864-020-06982-4

**Published:** 2020-12-16

**Authors:** Andrew Melnyk, Sergey Knyazev, Fredrik Vannberg, Leonid Bunimovich, Pavel Skums, Alex Zelikovsky

**Affiliations:** 1 Computer Science Department, Georgia State University, 25 Park Place NE, Atlanta, GA 30303 USA; 2 Georgia Institute of Technology, North Ave NW, Atlanta, GA 30332 USA; 3 I.M. Sechenov First Moscow State Medical University, Moscow, 119991 Russia

**Keywords:** Genetic relatedness, Transmission networks, Outbreaks investigations, K-mers, De Bruijn graph

## Abstract

**Background:**

RNA viruses mutate at extremely high rates, forming an intra-host viral population of closely related variants, which allows them to evade the host’s immune system and makes them particularly dangerous. Viral outbreaks pose a significant threat for public health, and, in order to deal with it, it is critical to infer transmission clusters, i.e., decide whether two viral samples belong to the same outbreak. Next-generation sequencing (NGS) can significantly help in tackling outbreak-related problems. While NGS data is first obtained as short reads, existing methods rely on assembled sequences. This requires reconstruction of the entire viral population, which is complicated, error-prone and time-consuming.

**Results:**

The experimental validation using sequencing data from HCV outbreaks shows that the proposed algorithm can successfully identify genetic relatedness between viral populations, infer transmission direction, transmission clusters and outbreak sources, as well as decide whether the source is present in the sequenced outbreak sample and identify it.

**Conclusions:**

Introduced algorithm allows to cluster genetically related samples, infer transmission directions and predict sources of outbreaks. Validation on experimental data demonstrated that algorithm is able to reconstruct various transmission characteristics. Advantage of the method is the ability to bypass cumbersome read assembly, thus eliminating the chance to introduce new errors, and saving processing time by allowing to use raw NGS reads.

## Background

RNA viruses mutate at extremely high rates, forming an intra-host viral population of closely related variants (or quasi-species). Their high variability [1] allows them to evade the host’s immune system and makes them particularly dangerous. Viral outbreaks pose a significant threat for public health, and, in order to deal with it, it is critical to infer transmission clusters, i.e., decide whether two viral samples belong to the same outbreak.

The progress of sequencing technologies made it possible to identify and sample intra-host viral populations at great depth [2–7]. Consequently, contribution of se- quencing technologies to molecular surveillance of viral outbreaks becomes more and more substantial. Genome sequencing of viral populations reveals similarities between samples, allows to measure viral genetic distance, and to facilitate outbreak identification and isolation. Computational methods can be used to infer transmis- sion characteristics from sequencing data. MiSeq [8] is a popuar NGS technology, that is used to sequence viral samples and detect rare viral mutations. Since MiSeq reads are short, their alignment and assembly for rapidly mutating RNA viruses is error-prone and complicated, which makes it appealing to develop an approach, that will allow to skip alignment and assembly steps.

In this paper, we apply an alignment- and assembly-free *k*-mer strategy to viral sequencing data. This strategy was initially introduced for analyzing NGS data in metagenomic studies, where reads come from multiple related and unrelated genomes (see [9]), as well as for RNA-seq quantification [10].

Indeed, it is relatively fast and easy to extract *k*-mers from reads, so that the complexity of viral distance measurement changes from read alignment and assem- bly to comparison of *k*-mer sets or distributions. Following [9], we build a De Bruijn graph for each sample, and then calculate Earth Mover’s Distance (EMD) between two *k*-mer distributions.

We applied the *k*-mer strategy to the following epidemiological tasks (T1-T5), where T1-T2 are applied to 2 hosts, and T3-T5 are applied to multiple hosts.

T1. **Identification of relatedness:**

**Given:** NGS reads from hosts *A* and *B*

**Decide:** Whether A and B are related (whether they belong to the same outbreak)

T2. **Identification of transmission direction:**

**Given:** NGS reads from hosts *A* and *B*

**Decide:** Whether host *A* infected *B* or *B* infected *A*

T3. **Identification of transmission clusters:**

**Given:** NGS reads from a set of hosts

**Find:** The transmission clusters corresponding to individual outbreaks

T4. **Presence of outbreak source:**

**Given:** NGS reads from a set of hosts

**Decide:** Whether outbreak source is present among sequenced hosts

T5. **Identification of outbreak source:**

**Given:** NGS reads from a set of hosts

**Find:** Outbreak source

Identifying whether 2 hosts belong to the same outbreak (T1) and transmission direction between them (T2) are tasks, that have to be solved in order to find trans- mission chains. Another important task is to discover boundaries of an outbreak (T3). Once hosts, that belong to an outbreak are obtained, it is critical to design whether the source is among them (T4). Finally, identifying the main spreader of an outbreak (T5) is a crucial epidemiological task, by solving which outbreak spreading can be prevented.

We experimentally validated our approach on a dataset, that consists of a collec- tion of HCV intra-host populations, sampled from 368 infected individuals [11].

Outbreak collection contains:

- 175 HCV samples from 34 epidemiologically curated outbreaks, reported to Centers for Disease Control and Prevention in 2008–2013. Outbreaks contain from 2 to 33 samples. Epidemiological histories, including sources of infection, are known for 11 outbreaks.
- Collection of 193 epidemiologically unrelated HCV samples.

Obtained results are comparable with existing approaches [11, 12], but proposed algorithm is much faster, since it doesn’t rely on read assembly.

## Methods

**Fig. 1:**
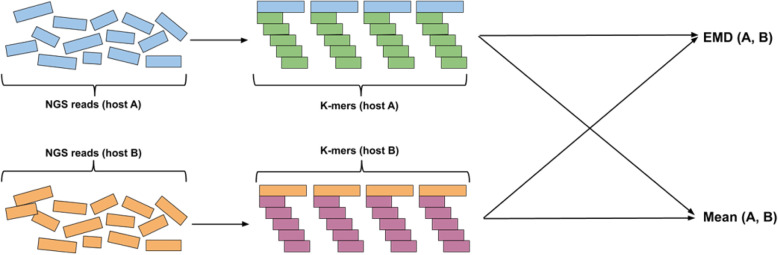
Algorithm pipeline. *k*-mer distributions for hosts, that need to be com- pared, are obtained from NGS reads. Then, EMD is computed and mean is obtained using *k*-mer distributions

Our algorithms are based on finding the distance between populations using *Earth Movers’ Distance (EMD)* between distributions of *k*-mers in NGS data. The general pipeline of the algorithm (see Fig. 1) includes obtaining *k*-mer distributions from NGS reads for corresponding hosts and computing EMD between them. As a result, we obtain mean of hosts *A* and *B Mean*(*A, B*) and EMD *EMD*(*A, B*) between them. We first describe how we find distances between *k*-mers and then describe how we find distance between samples.

### Finding distances between k-mers in the De Bruijn graph

*k-mer* refers to a substring of length *k*. In our work, we use *De Bruijn graph* to calculate distance between k-mers. De Bruijn graph is the graph, that is constructed so that vertices represent every string over a finite alphabet of length *l*, and edges are added between vertices that have overlap of *l* − 1.

Once De Bruijn graph is constructed, distance between k-mers can be calculated as a length of shortest path between corresponding vertices using *breadth-first search* algorithm. In our algorithms, obtained graph is converted to undirected be- fore shortest path computation.

### Finding EMD between viral samples

Viral populations can be compared by comparing the corresponding *k*-mer distri- butions using EMD. First, *k*-mer distributions are obtained for each sample, so that they contain all *k*-mers and normalized frequencies.

EMD is a method, that allows to evaluate dissimilarity between two multi- dimensional distributions in some feature space where a distance measure between single features (*ground distance*) is given [13]. Distributions can be represented as *signatures* - sets of clusters, so that each cluster is represented by its mean and by the fraction of distribution that belongs to that cluster. Computation of EMD is based on solving the *transportation problem*, which can be formulated as following: for several suppliers, each with a given amount of goods, several consumers, each with limited capacity, and a cost of transporting a single unit of goods between each supplier-consumer pair, find a least-expensive flow of goods from the suppliers to the consumers that satisfies the consumers’ demand. EMD is calculated as the following: $EMD\left(P,Q\right)={\varSigma}_{i=1}^m{\varSigma}_{j=1}^n{f}_{ij}{d}_{ij}$ where *f*_*ij*_ is the minimum-cost flow between supplier *i* and consumer *j*, and *d*_*ij*_ is the distance between *i* and *j*.

It should also be noted that EMD is usually normalized by the total flow, but we perform but we perform normalization of frequencies in k-mer distributions before EMD computation, which results in total flow always being equal to 1.

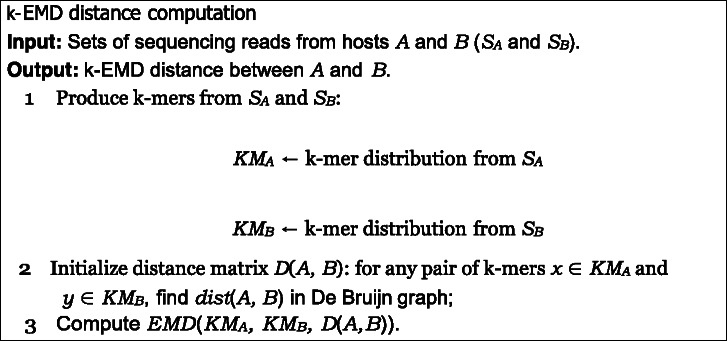



### Example of EMD computation

**Fig. 2:**
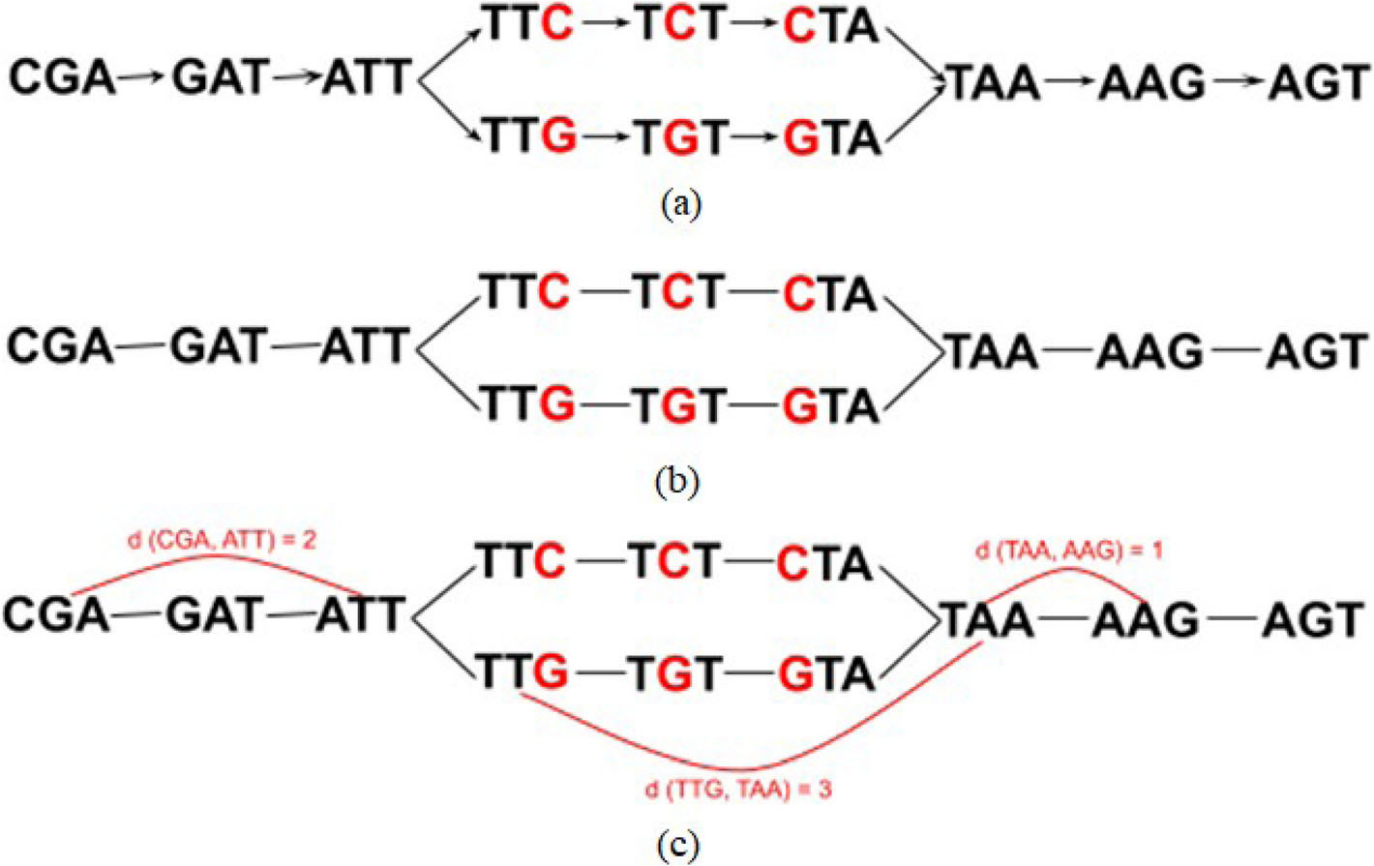
De Bruijn graph for 3-mers, obtained from sequences *CGATTCTAAGT* and *CGATTGTAAGT* . Once original graph is obtained (**a**), directions (**b**) are re- moved and pairwise distances are computed for all k-mers (**c**)

**Fig. 3:**
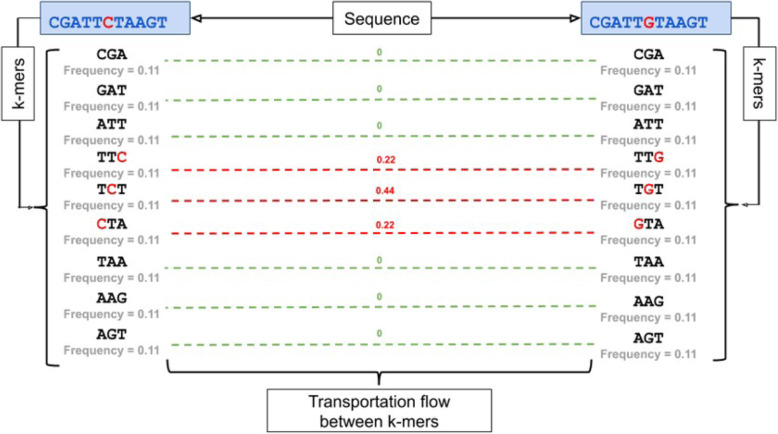
Finding EMD distance between *k*-mers of sequences *CGATTCTAAG* and *CGATTGTAAGT* . The *k*-mer distributions are on the left and right sides. Dashed lines represent transportation flow between k-mers; corresponding flow val- ues are shown in green. Red values on top of the lines represent distance between corresponding k-mers in the De Bruijn graph

Constructing of the De Bruijn graph between two sequences *CGATTCTAAGT* and *CGATTGTAAGT* is shown on Fig. 2. Once original graph is obtained, directions are removed and pairwise distances are computed for all k-mers. Figure 3 describes an example of k-EMD distance computation. After k-mer distributions are generated for input sequences, EMD is computed as the work, where *f*_*ij*_ is the flow between histogram(k-mer distribution) elements *i* and *j* and *d*_*ij*_ is the corresponding distance between k-mers, which is obtained from De Bruijn graph (Fig. 2). This way, *EMD* = 0*.*88.

### Mean *k*-mer distribution

Representing samples as k-mer distributions allows to estimate the center from a group of samples by introducing a mean host. We use the **maximum mean** *k*-mer distribution, which is obtained by finding the maximum observed frequency for each.

$$ \mathrm{k}-\mathrm{mer}\ {k}_i{f}_i^{max}={\mathit{\max}}_{1\le i\le n}{f}_i\ \mathrm{and}\ \mathrm{normalization}\ {f}_j^{\prime }=\frac{f_i^{max}}{\sum \limits_{1\le i\le n}{f}_i^{max}}. $$

### Identification of relatedness

We train out algorithm on all given outbreaks and obtain minimal EMD between 2 unrelated hosts, which we use as a threshold *t*. To identify whether 2 hosts *A* and *B* are related, we compute EMD between them *EMD*(*A, B*) and predict that they are related if *EMD*(*A, B*) *< t*, and unrelated otherwise.

### .Identification of transmission direction between hosts

To infer transmission direction between a pair of samples *X* and *Y*, we first compute a mean host *Mean*(*A, B*).

**Fig. 4:**
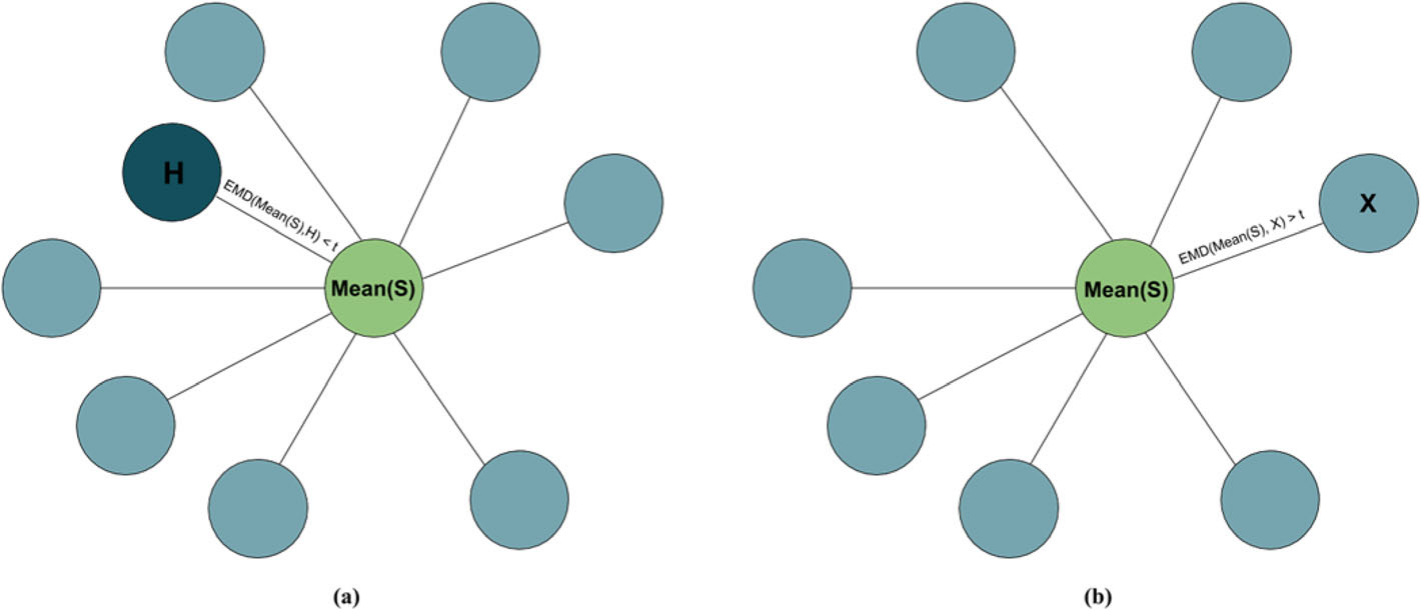
Inference of transmission between hosts *A* and *B*. First, mean host *Mean*(*A, B*) is introduced. Then *EMD* is computed between *Mean*(*A, B*) and hosts *A* and *B*. Finally, *EMD*(*Mean*(*A, B*)*, A*) is compared with *EMD*(*Mean*(*A, B*)*, B*). If *EMD*(*Mean*(*A, B*)*, A*) *< EMD*(*Mean*(*A, B*)*, B*), then transmission direction is predicted as the one that happened from *A* to *B*

Once *Mean*(*A, B*) is obtained, we calculate EMD between mean host and hosts *A* and *B EMD*(*Mean*(*A, B*)*, A*) and *EMD*(*Mean*(*A, B*)*, B*). Host, that is closer to the maximum mean is assumed to be the transmission source, so that if *EMD*(*Mean*(*A, B*)*, A*) *< EMD*(*Mean*(*A, B*)*, B*), we predict that the transmis- sion happened from *A* to *B* (Fig. 4).

### Identification of transmission clusters

To test hierarchical clustering, *single-linkage* algorithm was used. This method eval- uates the similarity of two clusters based on their most similar members [14] and groups clusters in bottom-up order until certain termination condition is satisfied. In our algorithm, we use a distance criteria, so clusters are merged until distance between them exceeds a pre-defined distance threshold, which represents EMD be- tween two closest unrelated samples in the dataset. This way, we obtain a partition, where some of the related hosts remain in different clusters. At this point, we pro- ceed to the second stage of the algorithm, that allows to improve the clustering quality by merging the clusters, that contain related hosts by performing the fol- lowing steps:

1. For each cluster, obtained from hierarchical clustering, compute center as the mean of all hosts within the cluster;
2. For each center, obtained at the previous step:

- Find distances to the furthest in-cluster host and closest host, that be- longs to the different cluster;
- If for cluster *A* there exists an ‘overlap’ (there is a host from cluster *B*, that is closer to the center than the furthest host, belonging to the same cluster (*A*)), merge *A* and *B*

**Fig. 5:**
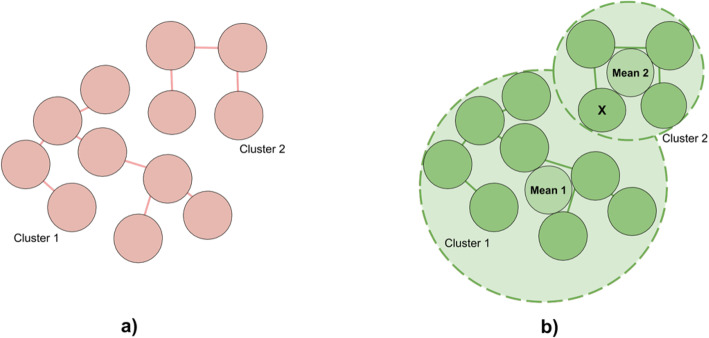
Example of overlap-based cluster merging. **a** Output of threshold-based hierarchical clustering, where circles represent hosts (k-mer distributions), that are connected with an edge if distance (EMD) between them doesn’t exceed a threshold (so that no unrelated hosts are connected). There are 2 clusters that belong to the same outbreak, which means that some related hosts are treated as unrelated. **b** For each cluster, circles were build, so that mean hosts reside in the center of the circle, and radius is defined as the distance between mean host and furthest host in an outbreak. Circle of cluster 1 intersects with cluster 2 since host X is closer to Mean 1 than furthest host in cluster 1. Therefore, clusters 1 and 2 are merged

Example of the algorithm is demonstrated in Fig. 5. a) shows output of threshold- based hierarchical clustering, where circles represent hosts, that are connected with an edge if distance between them doesn’t exceed a threshold. There are 2 clusters that belong to the same outbreak. b) shows how clusters are merged based on circle overlap. For each cluster, mean host of all hosts within the cluster is calculated (shown in the center). Circles with dashed borders have centers in respective mean hosts; their radiuses are calculated as distances between mean hosts and furthest in-cluster hosts. In the example, Mean 1 is closer to host *A* that to the furthest host from the same (left) cluster. This way, according to our algorithm, intersecting clusters collapse.

### Deciding whether source is present in a set of hosts

To decide whether source is present in a set of sequenced hosts *S*, the following algorithm is applied:

1. Calculate mean *Mean*(*S*) for all hosts within an outbreak;
2. For every host *H*, calculate EMD between *H* and mean *Mean EMD*(*Mean*(*S*)*, H*);
3. If there exists a host, for which *EMD*(*Mean*(*S*)*, H*) *< t*, source is present. To obtain threshold *t*, we train the algorithm on all outbreaks with known sources. For every such outbreak, we first calculate the mean host *Mean*(*S*) and distances between mean and every host *H* in the outbreak *EMD*(*Mean*(*S*)*, H*), find the smallest distance and normalize it by the median distance from mean to host in an outbreak. After this, we repeat the procedure for the same outbreak, but discard the source. We define *t* as the minimal *EMD*(*Mean*(*S*)*, H*) for an outbreak without source, which maximizes accuracy, so that outbreaks, where source is present, have *EMD*(*Mean*(*S*)*, H*) *< t*.

### Source identification

To identify sources, we find a maximum mean host for an outbreak *Mean* and cal- culate EMD between every host and *Mean*. Host with minimum *EMD*(*H, Mean*) is assumed to be the source.

### Runtime complexity

The algorithm uses Pele and Werman’s [15, 16] algorithm for fast EMD computation, which has a runtime complexity of *O*(*N* ^2^*UlogN*), where *N* is the number of nodes (k-mers), and *U* is an upper bound on the largest supply (flow) of any node (since frequencies are normalized, this is equal to 1). This way, *k* – *mer EMD* has a worst time complexity of *O*(*N* ^2^*logN*).

## Results

**Fig. 6:**
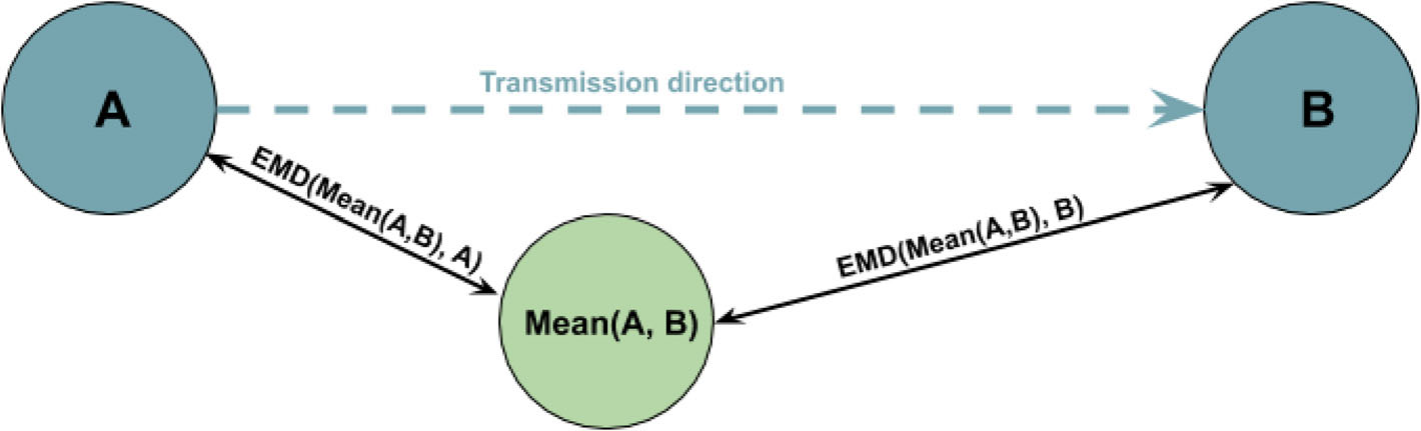
Deciding whether source is present in a given set of hosts. Here, every circle represents a host, belonging to an outbreak, and green circle represents mean. Edges represent distances between mean hosts and hosts in an outbreak. If there is a host, that is close to mean (so that the distance is smaller than a threshold, case (**a**)), we conclude, that source is present in an outbreak. Otherwise, analyzed set of hosts doesn’t include the outbreak source (case (**b**))

We validated our new algorithm on a publicly available dataset obtained from an epidemiological study of HCV outbreaks [11] Fig. 6.

### Data sets

The data consists of 368 sequenced hosts where 175 of them belong to 34 annotated outbreaks. Among these annotated outbreaks, 11 have a known main spreader (Table 1). All outbreaks contain from 2 to 33 hosts. Every host is represented as an HCV intra-host population, obtained with end-point limiting-dilution (EPLD). All viral sequences represent a fragment of E1/E2 genomic region of length 264 bp. Data samples annotation consists of host and outbreak id along with abundance for every sequence. This way, we were able to interpret obtained experimental results.

**Table 1:** Outbreaks with known sources

Outbreak	AA	AC	AI	AJ	AQ	AW	BA	BB	BC	BJ	NH
**# samples**	3	4	15	3	9	19	6	7	2	4	33

We simulated MiSeq reads from known haplotypes by SimSeq [17] and created mixtures using abundances from original data.

### Validation

#### Identification of relatedness

**Fig. 7:**
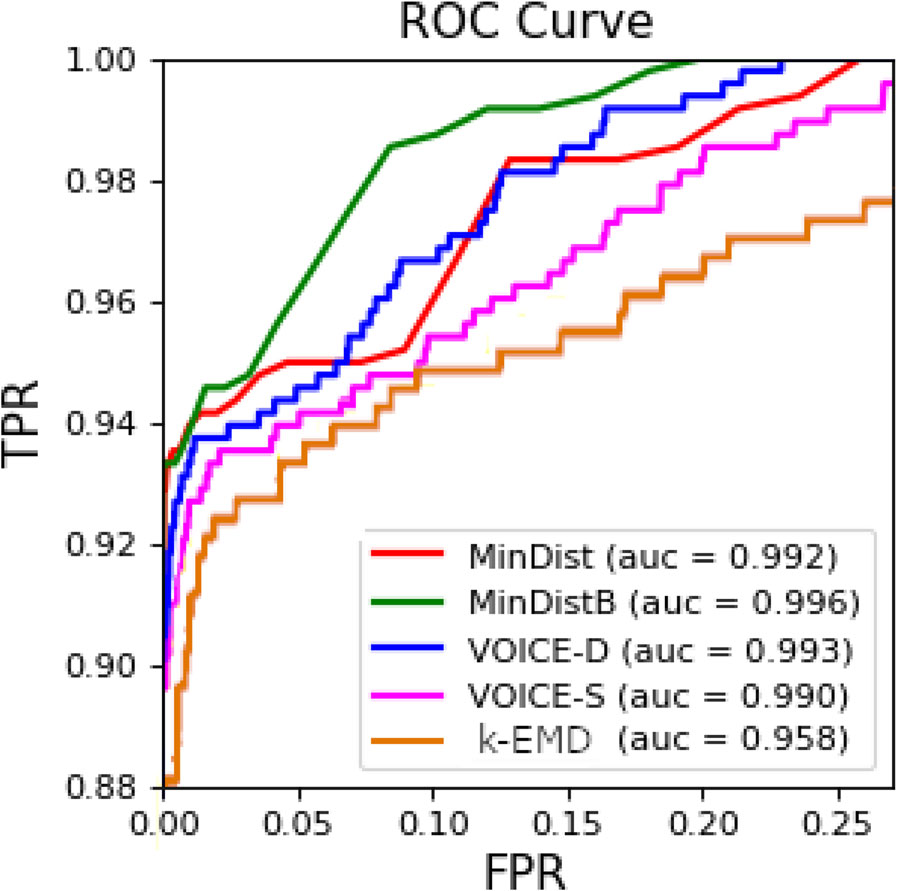
ROC curve for prediction of source presence. AUROC = 0.8

Viral populations from two samples are genetically related if they belong to the same outbreak and unrelated, otherwise. The genetic relatedness is validated on the union of both collections containing all outbreaks and unrelated samples. There are 67,528 host pairs (obtained from all 368 hosts). Among these pairs, 1007 represent related cases (so that both hosts in pair belong to the same annotated outbreak). We used EMD as predictor for relatedness. We measured the sensitivity of our method as following. First we determining the EMD value for all unrelated pairs, the mini- mum value we have chosen as a threshold which prohibits false-positive relatedness detection, the pairs which have EMD below the threshold are considered as related. Precision of our algorithm is 100%. We calculated the recall as a proportion of cor- rectly predicted related pairs among all known related pairs. Results are described in Table 2. Relatedness ROC is shown on Fig. 7.

**Table 2:** Validation results. k-EMD was tested on a dataset, that includes 34 out- breaks; MinDist, ReD and VOICE were validated earlier on a smaller dataset, that didn’t include one of the outbreaks. For convenience, results for k-EMD contain 2 values - one for the smaller dataset, and one for the entire (34 outbreaks) set of hosts (values in parentheses)

Method	k-EMD	MinDist	MinDistB	ReD	VOICE-D	VOICE-S
**Relatedness sensitivity, %**	80.4 (90)	90	92.9	55.3	85.2	86.8
**Clustering sensitivity, %**	100 (100)	100	100	96.3	98.2	98.2
**Direction accuracy, %**	88.7 (90.4)	N/A	N/A	87.1	83.9	87.1
**Source accuracy, %**	80 (81.8)	50	40	90	80	90

#### Identification of transmission direction between hosts

Performance of algorithm when identifying transmission direction was calculated as a ratio of pairs of hosts with correctly predicted directions to all host pairs, where direction is known. Results are shown in Table 2.

#### Identification of transmission clusters

Precision for our algorithm is equal to 100%, since we don’t merge hosts from different outbreaks. Similarities between true and estimated partitions were evalu- ated using an editing metric [18]. Given metric is defined as the minimum number of elementary operations, required to transform one partition into another, such as joining or partition of clusters [18]. Clustering recall was calculated similarly to [12], so that editing distance *E* was normalized by dividing it by the number of elementary operations *N*, required to transform trivial partition into singleton sets into true partition, which is equal to *n* − *k*, where *n* is the number of samples and *k* is the number of true clusters [12]:

$$ Recall=\frac{E}{n-k}\times 100\% $$

#### Deciding whether outbreak source is present

**Fig. 8:**
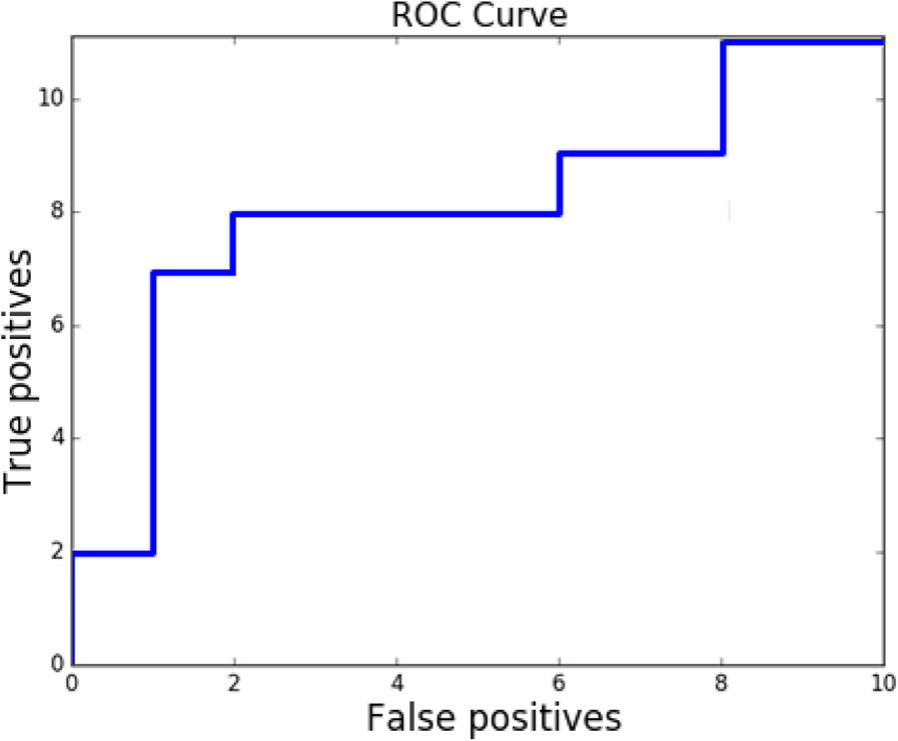
Relatedness prediction ROC curve for analyzed methods

Source presence recall was calculated as the proportion of outbreaks with present source, that were correctly identified as such; precision - as the proportion of cor- rectly identified outbreaks, where source is not present. Finally, specificity was cal- culated as the total number of outbreaks with present source, divided by the sum of total number of outbreaks with present source and the number of outbreaks, that were incorrectly identified to have a source present. For our algorithm, precision = 90%, specificity = 80%, and recall = 85%. ROC curve for source presence detection is shown on Fig. 8.

### Identification of outbreak sources

**Fig. 9:**
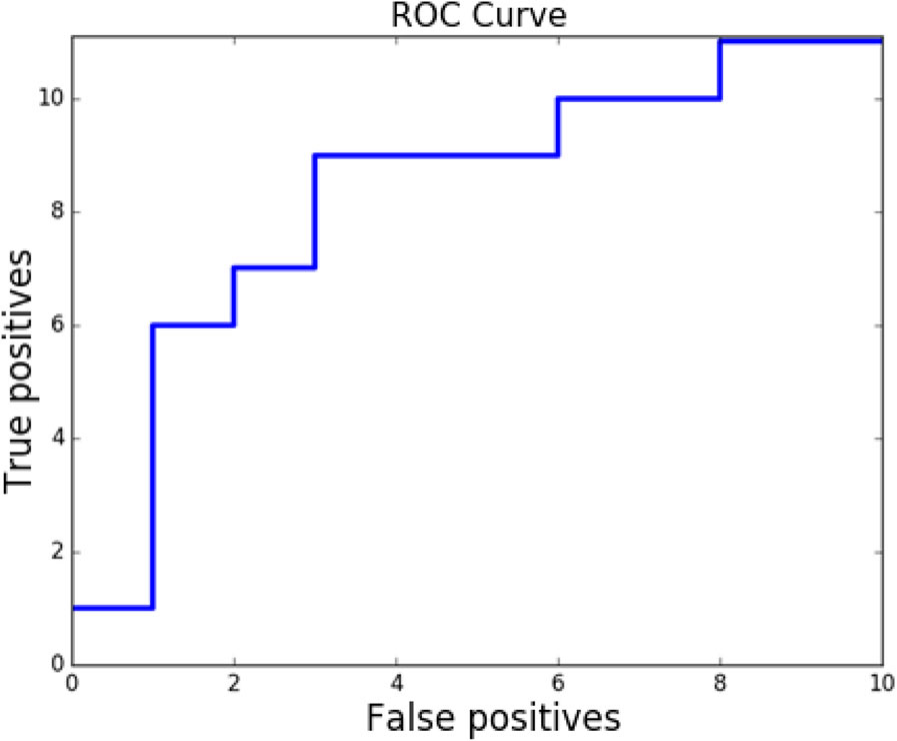
k-EMD ROC curve for source prediction. AUROC = 0.72

Source identification accuracy is calculated as the percentage of outbreaks with correctly predicted sources for outbreaks with known sources. ROC curve for source presence detection is shown on Fig. 9.

## Conclusions

Extracting haplotypes by EPLD is laborious and costly procedure and that pro- hibits previously developed methods [12] from wide spread. On the other hand, viral samples can be easily sequenced by NGS, and that makes our novel method attractive. Furthermore, we can see that results in this article are comparable with those which were obtained using EPLD technology [12]. Moreover, our method al- lowed to decide whether the spreader get sequenced.

Application of molecular viral analysis to investigation of outbreaks and infer- ence of transmission networks is a promising technique, that is available nowadays. However, it generates novel computational challenges. Given work introduced an algorithm for investigation of viral transmissions, that is based on analysis of the intra-host viral populations through k-mer decomposition. Proposed approach al- lows to cluster genetically related samples, infer transmission directions and predict sources of outbreaks. Validation on experimental data demonstrated that algorithm is able to reconstruct various transmission characteristics. It should be noted that even though there is still room for improvement when it comes to algorithm perfor- mance, advantage of the method is the ability to bypass cumbersome read assembly, thus eliminating the chance to introduce new errors, and saving processing time by allowing to use raw NGS reads.

## Data Availability

*k − mer EMD* is freely available at https://github.com/amelnyk34/kemd.

## Abbreviations

NGS: Next-generation sequencing
EMD: Earth mover’s distance
HCV: Hepatitis C virus
RNA: Ribonucleic acid
EPLD: End-point limiting-dilution
ROC: Receiver operating characteristic
